# Host Range and Symbiotic Effectiveness of N_2_O Reducing *Bradyrhizobium* Strains

**DOI:** 10.3389/fmicb.2019.02746

**Published:** 2019-11-29

**Authors:** Kedir Woliy, Tulu Degefu, Åsa Frostegård

**Affiliations:** ^1^Faculty of Chemistry, Biotechnology and Food Science, Norwegian University of Life Sciences, Ås, Norway; ^2^International Crops Research Institute for the Semi-Arid Tropics, Addis Ababa, Ethiopia

**Keywords:** *Bradyrhizobium*, denitrification, symbiotic nitrogen fixation, nitrous oxide, nitrous oxide reductase

## Abstract

Emissions of the potent greenhouse gas N_2_O is one of the environmental problems associated with intensive use of synthetic N fertilizers, and novel N_2_O mitigation strategies are needed to minimize fertilizer applications and N_2_O release without affecting agricultural efficiencies. Increased incorporation of legume crops in agricultural practices offers a sustainable alternative. Legumes, in their symbiosis with nitrogen fixing bacteria, rhizobia, reduce the need for fertilizers and also respond to the need for increased production of plant-based proteins. Not all combinations of rhizobia and legumes result in efficient nitrogen fixation, and legume crops therefore often need to be inoculated with compatible rhizobial strains. Recent research has demonstrated that some rhizobia are also very efficient N_2_O reducers. Several nutritionally and economically important legumes form root nodules in symbiosis with bacteria belonging to *Bradyrhizobium*. Here, the host-ranges of fourteen N_2_O reducing *Bradyrhizobium* strains were tested on six legume hosts; cowpea, groundnut, mung bean, haricot bean, soybean, and alfalfa. The plants were grown for 35 days in pots in sterile sand supplemented with N-free nutrient solution. Cowpea was the most promiscuous host nodulated by all test strains, followed by groundnut (11 strains) and mungbean (4 strains). Three test strains were able to nodulate all these three legumes, while none nodulated the other three hosts. For cowpea, five strains increased the shoot dry weight and ten strains the shoot nitrogen content (pairwise comparison; *p* < 0.05). For groundnut the corresponding results were three and nine strains. The symbiotic effectiveness for the different strains ranged from 45 to 98% in cowpea and 34 to 95% in groundnut, relative to fertilized controls. The N_2_O reduction capacity of detached nodules from cowpea plants inoculated with one of these strains confirmed active N_2_O reduction inside the nodules. When released from senescent nodules such strains are expected to also act as sinks for N_2_O produced by denitrifying organisms in the soil microbial community. Our strategy to search among known N_2_O-reducing *Bradyrhizobium* strains for their N_2_-fixation effectiveness successfully identified several strains which can potentially be used for the production of legume inoculants with the dual capacities of efficacious N_2_-fixation and N_2_O reduction.

## Introduction

Synthetic fertilizers are the major sources of fixed N in most agricultural systems and have contributed to boosting crop productivity since the beginning of the green revolution (Kox and Jetten, 2015). Excessive use of fertilizer, however, comes with environmental risks and has created problems such as pollution of water bodies and emission of nitrous oxide (N_2_O), a potent greenhouse gas and destructor of the stratospheric ozone layer (Ravishankara et al., 2009; Montzka et al., 2011; Reay et al., 2012; Butterbach-Bahl et al., 2013). Agriculture is a major source of N_2_O, and it has been estimated that more than 60% of the N_2_O emitted into the atmosphere is derived from N fertilized agricultural soils, mainly through the microbial processes of denitrification and nitrification (Thomson et al., 2012). Biological N_2_ fixation is an environmentally friendly alternative to synthetic fertilizers (Jensen and Hauggaard-Nielsen, 2003). Legumes in symbiosis with N_2_ fixing bacteria (rhizobia) account for the major part of the biological N_2_ fixation, with rates in different agricultural systems being about 115 kg N ha^–1^ year^–1^ for crop legumes and 110–227 for pasture and fodder legumes (Herridge et al., 2008). In comparison, other endophytic and free-living bacteria fixed <5 to 25 kg N ha^–1^ year^–1^. Thus, the incorporation of legumes in pastures and in crop rotation practices, or intercropping legumes with other plants, will lessen the need for synthetic N fertilizers (Dakora et al., 1987; Peoples et al., 2009; Jensen et al., 2012) and thereby contribute in the battle against N_2_O emissions.

The establishment of the legume-rhizobium symbiosis is based on a highly specialized signaling system, leading to the development of root nodules in the plant cortex. In most cases, rhizobia gain entrance to the plant via an infection thread formed in the root hairs, but in some plants, for example groundnut, the rhizobia instead enter through wounds in the root. The rhizobia then enter the cytoplasm of plant cells in the nodule where they, surrounded by plant-derived membranes, will differentiate into bacteroids. These are specialized bacterial cells that fix N_2_ into NH_3_, which is used by the plant for the synthesis of amino acids and proteins (Poole et al., 2018). Rhizobia vary in the type of hosts they nodulate. Some strains are highly specific, nodulating only a limited number of hosts, while others are promiscuous with a wide host range (Pueppke and Broughton, 1999). Moreover, some combinations of plant–rhizobia are efficient, others not, and the nitrogen fixation effectiviness may vary even between closely related plant cultivars/bacterial strains. Since soils may lack matching and/or effective rhizobia for certain legumes, especially when newly introduced to an area (Rodríguez-Echeverría et al., 2012), seeds of many legume crops need to be inoculated with compatible rhizobia. Strain screening and selection is an important step when developing new inoculants that are better specialized for selected crops and agricultural regions, aiming to optimize yields and plant nitrogen content.

Another feature of some rhizobia is their ability to denitrify, i.e., use nitrogen oxides as electron acceptors for respiration under anoxic conditions. Rhizobia with a complete denitrification pathway carry genes coding for the periplasmic nitrate reductase NapAB, the copper (Cu) containing nitrite reductase NirK, a cytochrome c-dependent nitric oxide reductase NorCB and a clade I NosZ (Bedmar et al., 2005; Sameshima-Saito et al., 2006; Torres et al., 2014; Mania et al., 2019). Genes for the membrane-bound NarG appear to be lacking (Mania et al., 2019), while a cd1-type nitrite reductase NirS has been reported in a few strains (Sánchez and Minamisawa, 2018). Denitrification has hitherto only been investigated in a limited number of rhizobia, and it is therefore not known how widespread this metabolism is in this group of organisms. Incomplete denitrification pathways lacking one or more of the reduction steps have been reported for strains of *Ensifer*, *Mesorhizobium*, and *Bradyrhizobium* (Falk et al., 2010; Bueno et al., 2015; Mania et al., 2019). Depending on if they carry and express the gene *nosZ*, coding for NosZ, different strains of rhizobia may serve either as sources or sinks for N_2_O emission. Thus, some rhizobia may have the potential to both enhance crop production and mitigate N_2_O emission, serving to counteract both problems of agricultural productivity and environmental risks associated with the use of synthetic N fertilizers. Among the N_2_O reducing rhizobia with such potentials are members of the genus *Bradyrhizobium*, of which many strains can form effective nodules with a wide range of economically important legume plants such as cowpea, peanut, soybean, and mungbean. Reports from greenhouse and field experiments demonstrate that inoculation of soybean with N_2_O-reducing strains of *Bradyrhizobium* can mitigate N_2_O emissions (Hénault and Revellin, 2011; Itakura et al., 2013; Akiyama et al., 2016). These findings are promising, and call for investigations of a wider range of rhizobia to identify N_2_O reducing strains that can be further investigated for their suitability as inoculants for different legumes.

In a recent study, we screened 39 *Bradyrhizobium* strains, isolated from legume trees and herbal crops in Ethiopia, and characterized them with respect to their taxonomy and phylogeny (Wolde-Meskel et al., 2004b, 2005). Among them, we found 20 *Bradyrhizobium* strains capable of denitrifying NO_3_^–^ to N_2_ (Mania et al., 2019). The phylogeny of these strains had been analyzed through multilocus sequence analyses (MLSA), which clustered them into seven genospecies (Degefu et al., 2017). The study by Mania et al. (2019) showed that 18 of the complete denitrifiers belonged to three different genospecies within the *Bradyrhizobium japonicum* superclade, while the other two belonged to the *Bradyrhizobium elkanii* superclade (genospecies defined by Degefu et al., 2017). When supplied with both NO_3_^–^ and N_2_O, these strains all showed a strong preference for reduction of N_2_O over NO_3_^–^, probably because the electron transport pathway to NosZ competes very efficiently for electrons compared to the pathway to NapAB (Mania et al., 2019). The strong capacity to reduce N_2_O was characteristic for all the complete denitrifying bradyrhizobia tested.

Aiming to find bradyrhizobia with the dual capacity of efficacious N_2_-fixation and N_2_O reduction, we here investigated the host ranges of 14 of these strongly N_2_O reducing *Bradyrhizobium* strains (Mania et al., 2019), all clustered in genospecies I (Degefu et al., 2017), against six legume hosts: cowpea (*Vigna unguiculata*), groundnut (*Arachis hypogea*), mung bean (*Vigna radiata*), soybean (*Glycine max*), haricot bean (*Phaseolus vulgaris*), and alfalfa (*Medicago sativa*). We also determined their symbiotic N_2_ fixation effectiveness in cowpea (12 strains) and groundnut (11 strains) and compared the symbiotic performance of six of the best performing strains with cowpea seeds collected from three locations in Ethiopia (Hawassa, Ziway, and Bale). Furthermore, we investigated the N_2_O reduction capacity of detached cowpea nodules formed by two different *Bradyrhizobium* strains, AC29c and AC70c. A major outcome of our study was that several of the N_2_O reducing *Bradyrhizobium* test strains fixed N_2_ effectively with different legume hosts. Such organisms are promising candidates for sustainable legume crop production and may simultaneously serve as a sink for N_2_O emission from the legume rhizosphere.

## Materials and Methods

### *Bradyrhizobium* Strains and Growth Conditions

Fourteen of the 39 *Bradyrhizobium* strains previously isolated from the tree and crop legumes growing in Ethiopia (Wolde-Meskel et al., 2004b) and capable of N_2_O reduction (Mania et al., 2019) were used (Table 1). Each strain was grown at 28°C for 6 days on yeast mannitol agar (YMA). A single colony was then aseptically picked and cultured to a cell density of ≈10^9^ cells ml^–1^ in a test tube containing 10 ml yeast mannitol broth (YMB) at 28°C. The YMA/YMB medium was as described in Mania et al. (2019).

**TABLE 1:** Host range of N_2_O reducing *Bradyrhizobium* strains tested on six legume crops.

**Strains^∗^**	**Host of isolation**	**Legume hosts tested**					
		
		**Cowpea^a^**	**Groundnut^b^**	**Mungbean^c^**	**Soybean^d^**	**Haricot bean^e^**	**Alfalfa^f^**
AC70c	*Phaseolus vulgaris*	√	√	√	–	–	–
AC79a	*Erythrina brucei*	√	–	–	–	–	–
AC79b1	*Erythrina brucei*	√	–	–	–	–	–
AC79b3	*Erythrina brucei*	√	√	–	–	–	–
AC79c2	*Erythrina brucei*	√	√	–	–	–	–
AC86b2	*Cajanus cajan*	√	√	–	–	–	–
AC86d2	*Cajanus cajan*	√	√	–	–	–	–
AC87h	*Millettia ferruginea*	√	√	–	–	–	–
AC87j1	*Millettia ferruginea*	√	√	–	–	–	–
AC87j2	*Millettia ferruginea*	√	√	–	–	–	–
AC92d	*Acacia gummifera*	√	√	–	–	–	–
AC101b	*Acacia saligna*	√	√	√	–	–	–
AC101c	*Acacia saligna*	√	√	√	–	–	–
AC101e	*Acacia saligna*	√	–	√	–	–	–

*The plants were harvested 35 days after inoculation, and host ranges were assessed by scoring the presence of nodules on the roots. ^∗^The phylogeny of 39 *Bradyrhizobium* strains isolated from legume plants in Ethiopia (Wolde-Meskel et al., 2004b) was determined through multilocus sequence analyses (MLSA) which clustered the strains into seven genospecies (Degefu et al., 2017). Here we tested the host range of 14 *Bradyrhizobium* strains in genospecies I, identified as “strong N_2_O reducers” (Mania et al., 2019). ^*a*^Local landrace Hawassa; ^*b*^local landrace from Gofa; ^*c*^local landrace from Gofa; ^*d*^variety Ethio Yugoslavia; ^*e*^variety Hawassa Dume; ^*f*^variety 1086.*

### Plant Growth, Host Range, and Symbiotic Effectiveness

The experiments were performed at Hawassa University, Ethiopia, in a polyhouse (a type of “shadehouse” with polyethylene roof and walls and fences to prevent animals from entering), at the temperature and humidity of the surrounding environment. We used six grain legume hosts: cowpea (local landrace, Hawassa, Ethiopia), groundnut (local landrace, Gofa, Ethiopia), mung bean (local landrace, Gofa), soybean (variety Ethio-Yugoslavia), haricot bean (variety Hawassa Dume) and alfalfa (variety 1086). Seeds were surface sterilized by immersion in 70% ethanol for 1 min, then transferred to a 3% sodium hypochlorite solution for 1 min followed by rinsing them in six changes of sterile water. Sterility was verified by streaking 2–3 seeds from each germination plate onto YMB agar. Sterilized seeds were imbibed in water for 1 h and subsequently incubated for germination at 28°C on sterile petri dishes lined with moist tissue paper for 3–4 days, or until the emergence of the radicle. Plants were grown in modified plastic cups with two separate parts: the lower part providing a reservoir for nutrient solution, and the upper part filled with washed and sterilized river sand, with a centrally positioned cotton wick extending through a hole out to the reservoir used as a rooting medium (Yates et al., 2016). Two germinated seeds were transferred to each plastic pot and thinned to one after successful seedling establishment. The experiments were carried out with three replicates using a randomized complete block design. The seedlings in each pot were inoculated with 1 ml (approximately 10^9^ cells ml^–1^) of rhizobial culture. Non-inoculated seedlings, either supplied with mineral nitrogen (as 0.05 g L^–1^ KNO_3_ weekly) or grown without nitrogen, were used as positive and negative controls, respectively. The seedlings inoculated with rhizobia were supplied with sterile, quarter-strength of Jensen’s modified N-free nutrient solution twice per week and with sterilized, distilled water as necessary (Somasegaran and Hoben, 1994).

Host ranges were assessed by scoring the number of root nodules after carefully uprooting the whole plant 35 days after inoculation. Symbiotic effectiveness of the strains was determined by measuring the nodule number (NN), nodule dry weight (NDW), and shoot dry weight (SDW) as described by Somasegaran and Hoben (1994). Plant shoot samples were oven-dried at 70°C for 48 h to determine the SDW, then ground to a fine powder to determine their nitrogen content using a LECO Truspec carbon, hydrogen and nitrogen analyzer (St. Joseph, MI, United States). The shoot nitrogen content is given as SN (%) of the sample dry weight according to the Dumas method (Nelson and Sommers, 1996). The symbiotic effectiveness (SE) was estimated as the percentage of SDW of the plants supplied with N (+N control) (Yates et al., 2016). Shoot nitrogen was estimated as percent N_2_ fixed from the atmosphere (Ndfa %): [(SN_inoculated_ − SN_uninoculated_)/SN_inoculated_] × 100. Since the plants were grown on sterile sand medium not supplied with an external source of nitrogen, the N accumulated in the plant shoots was assumed to be derived from the atmosphere through symbiotic N_2_ fixation. The shoot nitrogen content of the uninoculated plants (SN_uninoculated_) was considered to be equal to the N content of the seeds (Rennie, 1984). Average values ± standard deviations (*n* = 3) are reported if not otherwise stated. Comparisons of the symbiotic effectiveness in the different treatments were done using analysis of variance (ANOVA) and Tukey’s HD test. Correlations between the different parameters for symbiotic effectiveness were determined by Pearson’s correlation coefficient.

### Symbiotic Effectiveness of the Best Performing Strains in Cowpea Plants

After the first round of experiments, the six *Bradyrhizobium* strains that performed best with the cowpea landraces collected from Hawassa (located about 175 miles south of the Ethiopian capital Addis Ababa) were tested against two more cowpea landraces collected from Ziway (100 miles south of Addis Ababa) and Bale (300 miles south-east of Addis Ababa). Procedures for determining the N_2_ fixation effectiveness and shoot N were as described previously.

### N_2_O Reduction Capacity by Intact Legume Nodules

We also investigated the capacity of bradyrhizobia to reduce N_2_O when living inside nodules. This experiment was performed in a temperature-controlled greenhouse facility at the Norwegian University of Life Sciences, Norway. To obtain nodules, cowpea seeds were sterilized and germinated as described above. Seedlings were planted in pots with vermiculite as rooting medium and inoculated with either the *Bradyrhizobium* strain AC29c, which is incapable of reducing N_2_O, or with the N_2_O-reducing *Bradyrhizobium* strain AC70c (Mania et al., 2019). Plants were uprooted 40 days after inoculation and nodules were cut from the roots and immediately washed with sterile distilled water. Equal numbers of healthy-looking nodules of approximately similar size were placed in three 120 ml sample vials. For each vial, nodules from three plants were pooled to obtain measurable N_2_O reduction rates, thus nine plants were used in total. The dry weight of the nodules in each vial was approximately 18.9 mg. The vials were sealed with gas-tight butyl rubber septa and flushed with Helium (He) gas for 180 s to remove other gases in the vials. After releasing the He overpressure, 5% O_2_ and about 400 ppm N_2_O were injected into the headspace. The N_2_O production/reduction capacity of the nodules was monitored using a robotic incubation system (Molstad et al., 2007).

## Results

### Host Range of *Bradyrhizobium* Strains Capable of N_2_O Reduction

Among the 14 tested strains, all formed pink, N_2_ fixing nodules on cowpea, while 11 strains nodulated groundnut and 4 strains nodulated mungbean. We identified three strains (AC70c, AC101b, and AC101c) that were able to nodulate all these three crops. None of the strains nodulated soybean, haricot bean or alfalfa. Uninoculated controls did not form any nodule in any of the treatments. A complete list of the strains, their host of isolation and the cross-inoculation results is given in Table 1.

### Symbiotic Effectiveness

Twelve of the fourteen strains that nodulated cowpea “Hawassa” and all eleven that nodulated groundnut were further tested for their N_2_-fixation effectiveness. The treatments resulted in significant differences in SDW and SN (%) for both plants (*p* < 0.001). Results of multiple pairwise comparisons using Tukey’s HD test at α = 0.05 showed that the SDW of cowpea plants nodulated by five of the twelve N_2_O reducing *Bradyrhizobium* strains was significantly higher than the SDW of the uninoculated –N controls (three strains at *p* < 0.001 and two strains at *p* < 0.05) (Table 2). There were large variations in SDW between replicates within the treatments with a coefficient of variation (CV) ranging between 4.0 and 56.6%. The SDW ranged from 280 ± 30 (standard deviation, *n* = 3) mg plant^–1^ to 570 ± 30 mg plant^–1^ with the corresponding SE of 45 and 91%, respectively. Similarly, groundnut plants nodulated by three out of the eleven N_2_O reducing *Bradyrhizobium* strains had significantly higher SDW (one strain at *p* < 0.001 and two strains at *p* < 0.05) than uninoculated –N controls (Table 4). The SDW of groundnut plants ranged from 328 ± 90 to 919 ± 110 mg plant^–1^ with SE of 34 and 95%, respectively (Table 4). There were also large variations in SDW between replicates of groundnut plants, with CV ranging between 5.4 and 47.9%. The highest SDW scores, and thus SE% values, were achieved for both cowpea and groundnut by inoculation with strain AC70c. For these combinations the SE values were 91 and 95%, respectively. This exceeded the symbiotic effectiveness significantly (*p* < 0.05) of three cowpea-nodulating strains and two groundnut-nodulating strains. For cowpea, strains AC101b and AC92d were also highly effective with SE ≥ 80% of the +N control. In total, 9 strains out of 12 that nodulated cowpea and 9 strains out of 11 that nodulated groundnut were rated from moderately to highly effective, with SDW exceeding 50% of the +N controls.

**TABLE 2:** Symbiotic performance of 12 N_2_O reducing *Bradyrhizobium* strains inoculated with cowpea was determined based on nodule number (NN), nodule dry weight (NDW), shoot dry weight (SDW), symbiotic effectiveness (SE, calculated as % relative to SDW of +N controls), shoot nitrogen content (SN, given as % of SDW), and shoot nitrogen derived from the atmosphere (Ndfa, calculated as % SN relative to −N controls).

**Treatment**	**NN**	**NDW (mg plant^–1^)**	**SDW (mg plant^–1^)**	**SE (%)**	**SN (%)**	**Ndfa (%)**
AC70c	44 ± 11^a^	93 ± 12^a^	570 ± 44^ab^	91	4.07 ± 0.37^abc^	76
AC101b	59 ± 20^a^	90 ± 26^a^	557 ± 116^ab^	89	4.24 ± 0.15^ab^	77
AC92d	31 ± 12^a^	107 ± 55^a^	500 ± 144^abc^	80	3.34 ± 0.68^abcd^	70
AC79c2	45 ± 13^a^	100 ± 00^a^	443 ± 29^abc^	71	2.99 ± 0.81^bcd^	66
AC101c	57 ± 37^a^	103 ± 12^a^	430 ± 56^abc^	67	2.62 ± 0.82^cd^	60
AC101e	29 ± 15^a^	70 ± 10^a^	393 ± 59^abcd^	63	3.81 ± 0.07^abc^	75
AC86d2	37 ± 4^a^	83 ± 21^a^	367 ± 32^bcd^	59	3.08 ± 0.21^abcd^	69
AC87H	40 ± 9^a^	83 ± 51^a^	363 ± 115^bcd^	58	2.23 ± 0.81^de^	52
AC79b3	46 ± 19^a^	60 ± 36^a^	323 ± 183^bcd^	52	3.45 ± 0.54^abcd^	72
AC86b2	27 ± 7^a^	70 ± 30^a^	297 ± 40^cd^	47	2.91 ± 0.10^bcd^	67
AC87j2	40 ± 17^a^	63 ± 15^a^	290 ± 69^cd^	46	2.54 ± 0.54^cd^	61
AC87j1	30 ± 13^a^	50 ± 17^a^	280 ± 35^cd^	45	2.10 ± 0.67^de^	50
+N control	na	na	627 ± 25^a^	na	4.57 ± 0.00^a^	na
−N control	na	na	147 ± 31^d^	na	0.97 ± 0.02^e^	na
Average	40 ± 17	81 ± 30	399 ± 147	64 ± 20	3.07 ± 1.03	66 ± 12
*F* statistics	1.16^ns^	1.23^ns^	7.3^∗∗∗^	3.69^∗∗^	10.45^∗∗∗^	2.55^∗^

*Average values are given (*n* = 3) ± standard deviation. +N control: fertilized uninoculated; −N: non-fertilized uninoculated. Average values in the same column, followed by the same letter, are not significantly different at the 5% probability level by Tukey’s test. na, not applicable; ns, non-significant; ^∗^significant at 0.05; ^∗∗^significant at 0.01; ^∗∗∗^significant at 0.001.*

The cowpea plants inoculated with our N_2_O reducing *Bradyrhizobium* strains (11 out of 12) accumulated significantly higher amounts of N in their shoots (*p* < 0.001) than the uninoculated control (Table 2). All cowpea plants inoculated with these strains were able to derive most of their shoot nitrogen from the atmosphere (Ndfa ≥ 50%) (Table 2). Similarly, groundnut plants inoculated with 9 out of 11 strains accumulated significantly higher levels of N in their shoots (*p* < 0.001) than the uninoculated control plants (Table 4). Unlike cowpea plants, most of the nodulated groundnut plants derived <50% of their shoot N from the atmosphere through symbiotic nitrogen fixation (Table 4).

The number of nodules (NN) produced by cowpea was 40 ± 17 plant^–1^, while the average NN for groundnut was 20 ± 13 (Tables 2, 4). There was no significant difference in NN between treatments of the same plant species (*p* > 0.05). However, NDW for groundnut differed significantly (*p* < 0.05) between treatments with different bacterial strains, ranging between 21 ± 18 and 111 ± 44 mg plant^–1^. For cowpea, the NDW ranged between 50 ± 17 to 107 ± 55 mg plant^–1^, but the difference between the treatments was not statistically significant.

The Pearson correlation coefficients of the different parameters of symbiotic effectiveness in cowpea and groundnut are presented in Tables 3, 5, respectively. Nodule number for cowpea was significantly correlated with NDW (*r* = 0.38, *p* < 0.05) and SDW (*r* = 0.51, *p* < 0.01). In groundnut, NN showed a significant positive correlation only with NDW (*r* = 0.66, *p* < 0.001). NDW showed a significant positive correlation with SDW both in cowpea (*r* = 0.65, *p* < 0.001) and groundnut (*r* = 0.48, *p* < 0.01). Neither NN nor NDW showed any significant correlation with SN (%) in both legumes (*p* > 0.05). However, SDW showed a significant positive correlation with SN (%) both in cowpea (*r* = 0.74, *p* < 0.001) and in groundnut (0.53, *p* < 0.01).

**TABLE 3:** Pearson correlation coefficient among parameters of symbiotic performance of cowpea inoculated with 12 N_2_O reducing *Bradyrhizobium* strains (Table 2).

	**NN**	**NDW**	**SDW**	**SE (%)**	**SN (%)**	**Ndfa (%)**
NN	1.00					
NDW	0.381^∗^	1.00				
SDW	0.510^∗∗^	0.648^∗∗∗^	1.00			
SE (%)	0.510^∗∗^	0.648^∗∗∗^	1.00^∗∗∗^	1.00		
SN (%)	0.305^ns^	0.147^ns^	0.736^∗∗∗^	0.548^∗∗∗^	1.00	
Ndfa (%)	0.327^ns^	0.235^ns^	0.456^∗∗^	0.456^∗∗^	0.936^∗∗∗^	1.00

*ns, non-significant; ^∗^significant at 0.05; ^∗∗^significant at 0.01; ^∗∗∗^significant at 0.001.*

**TABLE 4:** Symbiotic performance of 11 N_2_O reducing *Bradyrhizobium* strains inoculated with groundnut was determined based on nodule number (NN), nodule dry weight (NDW), shoot dry weight (SDW), symbiotic effectiveness (SE, calculated as% relative to SDW of +N controls), shoot nitrogen content (SN, given as% of SDW), and shoot nitrogen derived from the atmosphere (Ndfa, calculated as% SN relative to −N controls).

**Treatment**	**NN**	**NDW (mg plant^–1^)**	**SDW (mg plant^–1^)**	**SE (%)**	**SN (%)**	**Ndfa (%)**
AC70c	24 ± 16^a^	83 ± 41^a^	919 ± 185^a^	95	1.9 ± 0.21^bc^	41
AC79b3	19 ± 10^a^	56 ± 9^a^	733 ± 207^ab^	76	1.8 ± 0.30^bc^	38
AC86b2	30 ± 4^a^	111 ± 44^a^	723 ± 244^ab^	75	1.9 ± 0.04^bc^	44
AC87j1	28 ± 15^a^	89 ± 7^a^	691 ± 165^abc^	72	1.6 ± 0.23^bc^	30
AC79c2	14 ± 8^a^	52 ± 20^a^	664 ± 60^abc^	69	1.9 ± 0.30^bc^	43
AC92d	16 ± 12^a^	56 ± 9^a^	651 ± 85^abc^	66	1.8 ± 0.14^bc^	39
AC101c	23 ± 18^a^	87 ± 65^a^	580 ± 134^abc^	60	1.9 ± 0.10^bc^	43
AC101b	10 ± 8^a^	21 ± 18^a^	576 ± 222^abc^	60	2.0 ± 0.33^bc^	44
AC86d2	28 ± 22^a^	57 ± 12^a^	564 ± 88^abc^	59	2.4 ± 0.05^b^	54
AC87H	15 ± 12^a^	37 ± 33^a^	423 ± 49^bc^	44	1.7 ± 0.23^cd^	35
AC87j2	10 ± 3^a^	24 ± 21^a^	328 ± 157^bc^	34	1.8 ± 0.27^cd^	38
+N control	na	na	965 ± 52^a^	na	3.1 ± 0.00^a^	na
−N control	na	na	277 ± 17^c^	na	1.1 ± 0.12^d^	na
Mean	20 ± 13	61 ± 38	623 ± 231	65 ± 21	1.91 ± 0.47	41 ± 9
*F* statistics	0.93^ns^	2.55^∗^	5.67^∗∗∗^	2.99^∗^	14.42^∗∗∗^	2.09^ns^

*Average values are given (*n* = 3) ± standard deviation. +N control: fertilized uninoculated; −N: non-fertilized uninoculated. Average values in the same column, followed by the same letter, are not significantly different at the 5% probability level by Tukey’s test. na, not applicable; ns, non-significant; ^∗^significant at 0.05; ^∗∗∗^significant at 0.001.*

**TABLE 5:** Pearson correlation coefficient among parameters of symbiotic performance of groundnut inoculated with 11 N_2_O reducing *Bradyrhizobium* strains (Table 4).

	**NN**	**NDW**	**SDW**	**SE (%)**	**SN (%)**	**Ndfa (%)**
NN	1.00					
NDW	0.657^∗∗∗^	1.00				
SDW	0.254^ns^	0.484^∗∗^	1.00			
SE (%)	0.254^ns^	0.484^∗∗^	1.00	1.00		
SN (%)	0.229^ns^	0.042^ns^	0.528^∗∗^	0.079^ns^	1.00	
Ndfa (%)	0.204^ns^	0.045^ns^	0.111^ns^	0.111^ns^	0.984^∗∗∗^	1.00

*ns, non-significant; ^∗∗^significant at 0.01; ^∗∗∗^significant at 0.001.*

### Relative Effectiveness of the Best Performing Strains in Three Cowpea Landraces

The six *Bradyrhizobium* strains that showed the best symbiotic performance with the cowpea landrace CPH (AC70c, AC101b, AC79c2, AC101c, AC101e, and AC92d) were also tested with landraces Cowpea Bale (CPB) and Cowpea Ziway (CPZ) (Table 6). The SDW and SN (%) values were higher to much higher than the −N control, but due to the variation between replicates, this was not statistically significant in most cases. Thus, unlike for CPH, no significant increase in SDW was found for CPZ and CPB after inoculation with the different strains (*p* > 0.05), except for strain AC79c2 with CPB (*p* < 0.05). Significant increases in SN (%) were, however, seen for CPZ inoculated with five of the strains (*p* < 0.001), and for CPB inoculated with two of the strains (*p* < 0.05). No significant differences were found when we compare the different strains with respect to their symbiotic performance within the same cowpea landrace. Comparison between individual strains, on the other hand, revealed differences in symbiotic effectiveness with the three landraces. Strain AC70c showed the best performance with CPH, resulting in the highest SDW recorded among the inoculated plants (570 ± 44 mg plant^–1^), while strain 101b performed best with CPZ, a combination that resulted in the highest SE (95% of the SDW of the +N control). Strain AC79c2 performed best with CPB. The shoot N content (given as % N of the SDW) also varied between different bacterial-plant combinations. The highest SN (%) recorded was in CPH inoculated with AC101b and AC70c, with values of 4.24 ± 0.15 and 4.07 ± 0.37%, respectively. All the CPH and CPZ plants derived most of their SN (%) from the atmosphere through symbiotic nitrogen fixation (Ndfa > 50%). Contrary to this, three strain-CPB combinations derived less than 50% of their shoot nitrogen from the atmosphere (Ndfa < 50%).

**TABLE 6:** Relative effectiveness of the six best performing *Bradyrhizobium* strains inoculated with two additional cowpea landraces Cowpea Ziway (CPZ) and Cowpea Bale (CPB), compared with Cowpea Hawassa (CPH) from first round of experiments.

**Treatment**	**SDW (mg plant^–1^)**			**SE (%)**			**SN (%)**			**Ndfa (%)**		
				
	**CPH**	**CPZ**	**CPB**	**CPH**	**CPZ**	**CPB**	**CPH**	**CPZ**	**CPB**	**CPH**	**CPZ**	**CPB**
AC70c	570 ± 44^ab^	350 ± 200^ab^	425 ± 35^ab^	91	73	77	4.07 ± 0.37^ab^	3.52 ± 0.61^a^	2.61 ± 0.02^ab^	76	71	50
AC101b	557 ± 116^ab^	457 ± 202^ab^	460 ± 28^ab^	89	95	84	4.24± 0.15^*ab*^	3.18 ± 0.44^a^	3.78 ± 0.30^a^	77	68	66
AC92d	500 ± 144^ab^	347± 159^*ab*^	495 ± 35^ab^	80	72	90	3.34 ± 0.68^a-c^	3.37 ± 0.57^a^	3.01 ± 0.88^ab^	70	70	55
AC79c2	443 ± 29^ab^	300± 139^*ab*^	540 ± 57^a^	71	63	98	3.00 ± 0.81^b−c^	3.23 ± 0.57^a^	2.41 ± 1.2^ab^	66	68	38
AC101c	430 ± 57^ab^	357± 109^*ab*^	450 ± 156^ab^	69	74	82	2.62 ± 0.82^c^	2.74 ± 0.69^a^	2.01 ± 0.38^ab^	60	62	34
AC101e	393 ± 59^b^	307 ± 75^ab^	490 ± 14^ab^	63	64	89	3.81 ± 0.07^a-c^	2.59 ± 0.98^ab^	2.38 ± 0.06^ab^	75	56	45
+N control	627 ± 25^a^	483 ± 104^a^	550 ± 42^a^	na	na	na	4.57 ± 0.00^a^	3.90 ± 0.00^a^	4.01 ± 0.00^a^	na	na	na
−N control	147 ± 31^c^	83 ± 6^b^	260 ± 57^b^	na	na	na	0.97 ± 0.02^d^	1.01 ± 0.00^b^	1.30 ± 0.00^b^	na	na	na
Average	458 ± 155	335 ± 164	458 ± 101	77 ± 15	73 ± 29	87 ± 11	3.33 ± 0.24	2.94 ± 0.20	2.67 ± 0.24	70 ± 9	65 ± 10	48 ± 15
*F* statistics	11.77^∗∗∗^	2.29^ns^	3.69^∗^	2.13^ns^	0.39^ns^	0.64^ns^	16.54^∗∗∗^	7.15^∗∗∗^	5.27^∗^	2.34^ns^	1.02^ns^	1.22^ns^

*Shoot dry weight (SDW), symbiotic effectiveness (SE, calculated as % relative to SDW of +N controls), shoot nitrogen content (SN, given as% of SDW) and shoot nitrogen derived from the atmosphere (Ndfa, calculated as % SN relative to −N controls). Average values are given (*n* = 3) ± standard deviation. +N control: fertilized uninoculated; −N: non-fertilized uninoculated. Average values in the same column, followed by the same letter, are not significantly different at the 5% probability level by Tukey’s test. na, not applicable; ns, non-significant; ^∗^significant at 0.05; ^∗∗∗^significant at 0.001. The means and levels of siginificance for each parameter were re-calculated for CPH relative to +N and −N controls.*

### N_2_O Reduction by Legume Nodules

**FIGURE 1:**
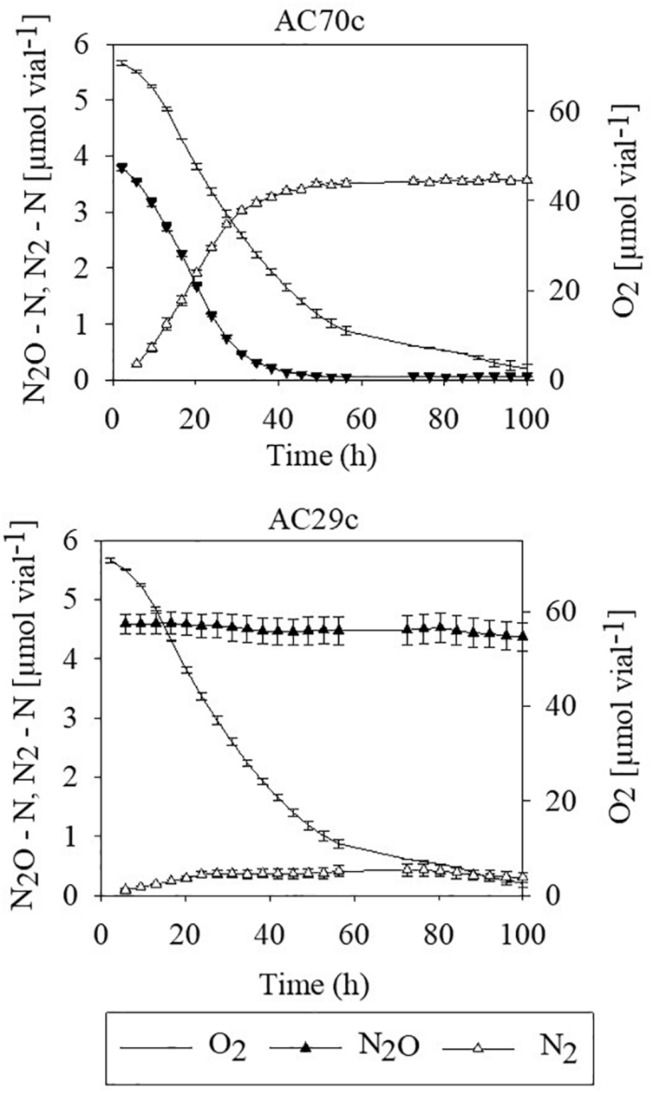
N_2_O reduction by detached nodules obtained from cowpea plants that had been inoculated with either the N_2_O reducing *Bradyrhizobium* strain AC70c, or with the non-N_2_O reducing *Bradyrhizobium* strain AC29c. The nodules (approximately 18.9 mg dry weight of nodules per vial) were incubated in vials containing He mixed with 5% O_2_ and 4–5 μmol N_2_O–N (corresponding to about 400–500 ppm N_2_O). *n* = 3; bars indicate standard deviation.

To evaluate whether bradyrhizobia inside nodules may contribute to the reduction of N_2_O, intact nodules collected from cowpea plants were placed in airtight vials containing He mixed with 5% O_2_ and 4–5 μmol N_2_O–N, and gas concentrations (O_2_, N_2_O, and N_2_) were monitored during 100 h (Figure 1). Nodules from plants inoculated with *Bradyrhizobium* strain AC70c, previously shown to reduce N_2_O when grown in batch culture (Mania et al., 2019), reduced exogenously supplied N_2_O to N_2_ at a rate of 6 μmol N g^–1^ nodules simultaneously with O_2_ consumption until all N_2_O was reduced to N_2_ (Figure 1). In contrast, nodules of cowpea plants inoculated with strain AC29c, classified as a phenotype lacking N_2_O reduction capacity (Mania et al., 2019) failed to reduce exogenously supplied N_2_O to N_2_. Cowpea nodules containing strain AC70c reduced exogenously supplied N_2_O to N_2_ within 40 h of incubation, unlike those containing strain AC29c which did not produce N_2_. The slight decrease in N_2_O in flasks containing nodules with strain AC29c is due to sampling loss.

## Discussion

Emissions of N_2_O are steadily increasing, and the International Panel of Climate Change foresees continued increases during the rest of the present century unless effective measures are undertaken (Rogelj et al., 2018). In accordance, Davidson and Kanter (2014) pointed out the need for novel mitigation options beynd “good agronomic practice” such as avoiding excessive use of fertilizers (Thomson et al., 2012). One such option could be to increase the production of nutrient-rich legumes, since this would reduce the needs for fertilizers and at the same time provide nutritious food and feed for people and livestock. Inoculation of legume crops with N_2_O reducing rhizobia, as suggested by, e.g., Hénault and Revellin (2011) and Itakura et al. (2013), would further enforce the N_2_O mitigation. The potential of this is emphasized by the recent findings that a range of taxonomically diverse bradyrhizobia exhibit very efficient N_2_O reduction, showing a strong preference for N_2_O over NO_3_^–^ as electron acceptor caused by the N_2_O reduction pathway being a powerful competitor for electrons compared to that of the periplasmic nitrate reductase Nap (Mania et al., 2019). This is likely to be a general trait common to all organisms, including rhizobia, that carry N_2_O reductase as well as Nap but lack the membrane-bound nitrate reductase Nar. Such organisms have the potential to act as sinks for N_2_O, produced in soil both by themselves and by other microorganisms.

Rhizobia vary in their compatibility as well as in their N_2_ fixation effectiveness (Musiyiwa et al., 2005). Consequently, an increased, yet sustainable, production of legume crops will require a wider variety of rhizobial inoculants, selected for providing the highest possible N_2_-fixation efficacy in combination with different types of legume crops, and at the same time carrying the capacity to reduce N_2_O. In a recent investigation of a wide diversity of *Bradyrhizobium* strains, we found that approximately 50% were able of N_2_O reduction (Mania et al., 2019), and in another collection of strains isolated mostly from nodules of peanut, only about one-third reduced N_2_O (Å. Frostegård, pers comm). The search for promising strains should focus on performing the time-consuming and laborious cross-inoculation tests on strains that are selected based on their ability to reduce N_2_O.

The 14 *Bradyrhizobium* strains studied here, all known to have a strong capacity for N_2_O reduction, nodulated at least one of the legume species, and some showed efficacious N_2_-fixation capacity (Tables 1, 2, 4). The results showed, however, clear compatibility differences between different combinations of test strain and the legume host. Both legume hosts and microsymbionts varied regarding their levels of promiscuity. Cowpea was the most promiscuous host and was nodulated by all the test strains, followed by peanut (11 strains) and mung bean (4 strains), while none of the strains nodulated soybean, haricot bean and alfalfa. This is in agreement with earlier reports showing that cowpea, groundnut and mungbean belong to the same cross-inoculation group (Somasegaran and Hoben, 1994) which can be nodulated by several *Bradyrhizobium* strains and are reported as promiscuous hosts (Peoples et al., 1989; Steenkamp et al., 2008; Pule-Meulenberg et al., 2010; Jaiswal et al., 2017; Ndungu et al., 2018). On the microsymbiont side, the three strains AC70c, AC101b, and AC101c that nodulated cowpea, groundnut, and mung bean may, therefore, be regarded as promiscuous.

Rhizobial strains with a wide host range and proven N_2_ fixation effectiveness are often preferred for the development of inoculants suitable for different legume crops (Drew et al., 2012). Except for strain AC70c, which was isolated from trap host species *P. vulgaris*, other test strains included in this study were isolated from tree legumes growing in Ethiopia (Wolde-Meskel et al., 2004b). This indicates that rhizobia either isolated from wild legumes or trapped from soil can be domesticated and used as inoculants for legume crops as reported earlier (Wange, 1989; Zhang et al., 1991). This potential can be further exploited as Ethiopia hosts a wide diversity of *Bradyrhizobium* strains in its soil (Aserse et al., 2012; Degefu et al., 2017, 2018) that can be used as inoculants for grain legumes. Our test strains failed to nodulate soybean, haricot bean, and alfalfa. This could be due to the specificity of these hosts to a certain group of rhizobia (Peoples et al., 1989). Reports show that alfalfa and haricot bean form a symbiotic association with fast-growing rhizobial strains belonging to genus *Ensifer* and *Rhizobium*, respectively, but not with bradyrhizobia (Somasegaran and Hoben, 1994). The inability of strain AC70c to nodulate haricot bean, its trap host (Wolde-Meskel et al., 2004a), could also be due to the specificity of the host genotype to a particular strain (Abi-Ghanem et al., 2011). We speculate that failure of our *Bradyrhizobium* test strains to nodulate soybean, a common host for *B. japonicum* strains, might be due to incompatibility as soybean is an exotic crop introduced to Ethiopia in the 1950s (Aserse et al., 2012). Classification of soybean under the cross-inoculation group different from cowpea and groundnut (Somasegaran and Hoben, 1994) further supports this.

Nodulation alone might not always result in an effective symbiosis that can fulfill the host’s N requirement (Terpolilli et al., 2008), necessitating careful screening and selection for elite rhizobial symbionts that can form effective N_2_ fixing nodules with the host legumes. The symbiotic effectiveness (SE) in our study ranged between 45 and 98% in the different cowpea landraces and between 34 and 95% in groundnut. Most of the test strains that nodulated both legume crops, however, were rated as moderately to highly effective with SDW exceeding 50% of the N fertilized controls, indicating their potential suitability as inoculants for cowpea and groundnut provided that they are competitive with other rhizobia in different soil environments. Inspection of the phylogenetic tree constructed based on the symbiosis-related gene *nodA* shows that most of our test strains are closely related to *Bradyrhizobium* strain CB756, a widely used inoculant of cowpea and peanut (Degefu et al., 2017), supporting their suitability as inoculants for the tested legumes. This should, however, be followed by field evaluation of the strain’s ability to compete with indigenous rhizobia for nodulation and adaptation to the conditions in the soil (moisture content, temperature, acidity, and others) that might limit the efficiency and survival of the inoculant (O’Hara et al., 2002). In cases where the soil harbors high numbers of less effective indigenous rhizobia, the selected strain should be both competitive for nodule occupancy and have a high N fixation effectiveness with the selected legume crop, to ensure the success of inoculation (Toro, 1996).

Among the most effective strains in the present study was strain AC70c that occupied its own distinct branch designated as C on the *nodA* phylogenetic tree (Degefu et al., 2017). It was the best performing strain both with cowpea and groundnut, with SE values of 98 and 95%, respectively. Differences in nodulation and symbiotic effectiveness of rhizobial strains can also be extended to genotypes of the same host legume species (Pazdernik et al., 1997; Abi-Ghanem et al., 2011; Yates et al., 2016). Our analysis of the relative effectiveness of the six best-performing strains on the three cowpea landraces (Table 6) also demonstrated such variations, confirming the need to evaluate the host range and symbiotic effectiveness of rhizobial strains with different cultivars of the same legume species.

Our results also showed that all the cowpea and groundnut plants inoculated by the test strains accumulated more N from the atmosphere through symbiotic N_2_ fixation than uninoculated non-fertilized controls. Cowpea plants derived most of their shoot N from the atmosphere through symbiotic N_2_ fixation. The low Ndfa in groundnut (<50% in most strain-plant combinations) might be due to the somewhat short duration of the experiment (35 days). Growth up to 7 weeks was recommended for large-seeded legumes such as groundnut before assessing nodulation and effectiveness of the inoculants (Yates et al., 2016).

Soybean nodules containing *Bradyrhizobium* strains have been reported to reduce N_2_O (Hirayama et al., 2011; Hénault and Revellin, 2011; Inaba et al., 2012). In accordance, our results showed that detached nodules from cowpea plants, inoculated with the N_2_O reducing strain AC70c (Mania et al., 2019), efficiently reduced exogenously provided N_2_O. This strain expressed N_2_O reductase *in vivo*, indicating the presence of bacterial cells with active anaerobic respiration in the root nodules. Yet, it is likely that the most important N_2_O reduction activity will be performed by N_2_O reductase expressing bradyrhizobia when they are released from senescent nodules into the soil where they can act as sinks for N_2_O produced by denitrifying organisms in the microbial community (Akiyama et al., 2016).

Taken together, through screening of several rhizobial strains for their N_2_O reduction capacity and N_2_ fixation effectiveness with different legume hosts, we obtained promising strains such as AC70c that can be used for the production of legume inoculants with both capacities. Such inoculants have the potential to increase the productivity of different legume crops and simultaneously mitigate N_2_O emission from agricultural fields.

## Data Availability Statement

The datasets generated for this study are available on request to the corresponding authors.

## Author Contributions

KW, TD, and ÅF planned the experiments, analyzed the results, and wrote the manuscript. KW and TD performed the experiments.

## Conflict of Interest

The authors declare that the research was conducted in the absence of any commercial or financial relationships that could be construed as a potential conflict of interest. The handling editor declared a past co-authorship with one of the authors, ÅF.
